# A case report of disseminated *Streptococcus pneumoniae infection* complicated by infective endocarditis, septic arthritis and epidural abscess in an immunocompetent patient

**DOI:** 10.1099/acmi.0.000611.v3

**Published:** 2023-07-17

**Authors:** Kimberley Rose Dean, Archana Koirala, Harsha Samarasekara

**Affiliations:** ^1^​ Resident Medical Officer, Orange Health Service of Western NSW Local Health District, 1530 Forest Road, Orange, NSW 2800, Australia; ^2^​ Staff Specialist in Immunology and Paediatrics, New South Wales Immunisation Specialist Service (NSWISS) Team, Nepean Blue Mountains Local Health District, Derby St., Kingswood, NSW 2747, Australia; ^3^​ Supervising Pathologist, Department of Microbiology, Pathology West-Orange, Orange Health Service, 1530 Forest Road, Orange, NSW 2800, Australia; ^4^​ Staff Specialist in Department of Pathology, Nepean Hospital of Nepean Blue Mountains Local Health District, Derby St, Kingswood NSW 2747, Australia

**Keywords:** bacteraemia, case study, infectious diseases, invasive pneumococcal disease, otitis media, pneumococcal, sepsis

## Abstract

*
Streptococcus pneumoniae* is a highly virulent, vaccine-preventable pathogen which can cause disease on a spectrum from benign to fatal. Apart from pneumonia, it commonly causes septicaemia and meningitis. This case report describes an unusual range of complications in a 53-year-old Caucasian female presenting to a regional hospital, without any risk known factors for severe disease (such as extremes of age, immunodeficiency or co-morbidities). Progressing from an episode of otitis media, her condition rapidly progressed to mastoid sinusitis, septic arthritis, infective endocarditis, epidural abscesses and multiple subcutaneous abscesses. Following quick identification of *S. pneumoniae* from a positive blood culture, the patient was treated with high-dose benzylpenicillin and ceftriaxone and aggressive source control by surgery, enabling a good clinical recovery.

## Data Summary

As this was a case study, no data sets were accessed for this article.

## Introduction

**Fig. 1.**
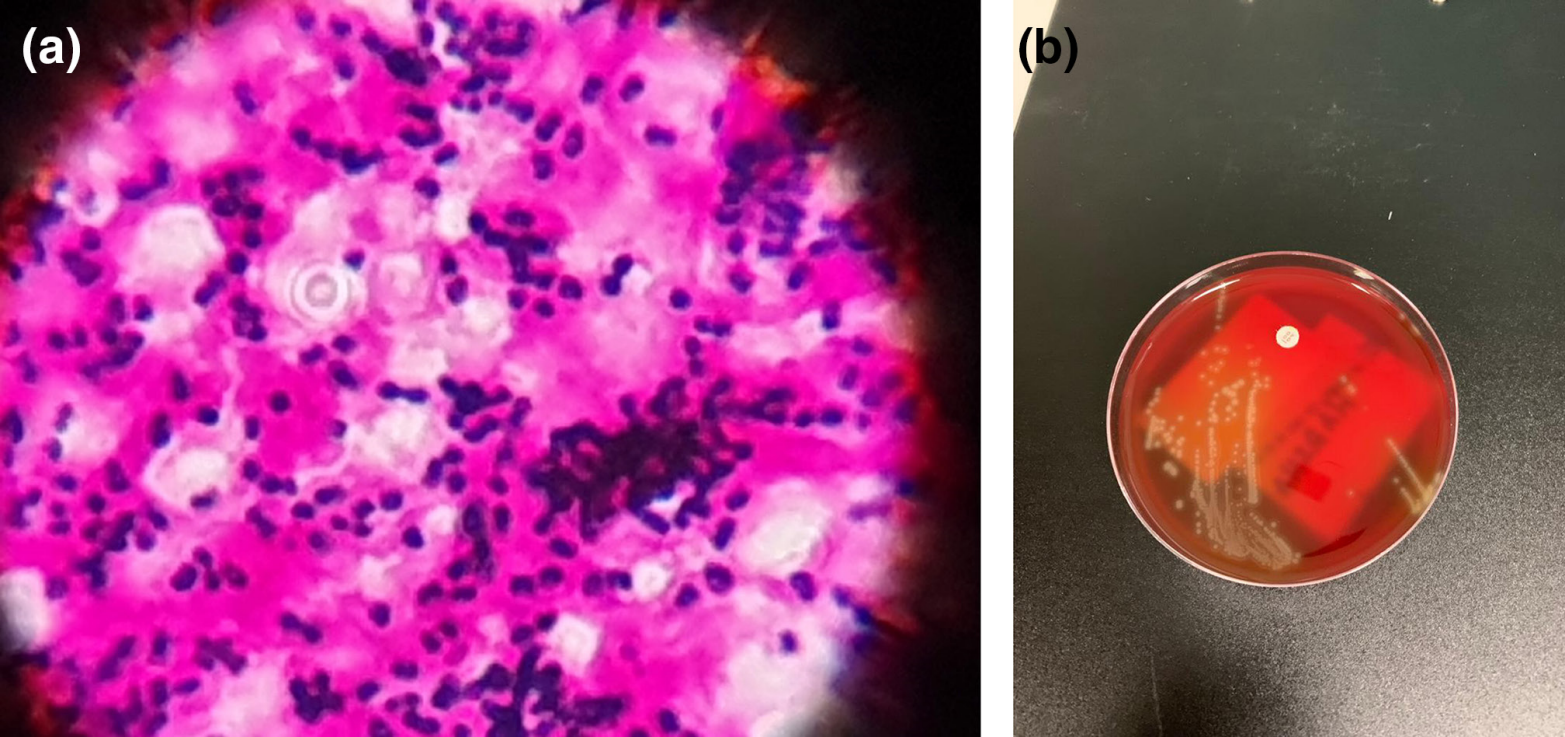
Gram stain (**a**) and culture plate (**b**) demonstrating *Streptococcus pneumoniae* with classical lancet shape, often in pair formation, indicating the culprit organism [53].

*Streptococcus pneumoniae* is a Gram-positive, alpha haemolytic *Streptococcus* with 97 known serotypes [1, 2] (Fig. 1). Invasive pneumococcal disease (IPD) is defined based on detection of *S. pneumoniae* by culture or nucleic acid testing from a normally sterile system; examples include pneumococcal meningitis and bacteraemia [3, 4]. *S. pneumoniae* accounts for 50 % of otitis media (OM) and is responsible for around 27 % of cases of pneumonia worldwide [5, 6].

In 2005, the pneumococcal vaccine was introduced in Australia and is part of the immunization schedule for all infants aged <2 years, with additional vaccines advised for at-risk individuals and older adults (Aboriginal and Torres Strait Islanders >50 years old and non-indigenous Australians >70 years of age). The two main vaccines offered are: 13-valent polysaccharide conjugate vaccine (13vPCV) and 23-valent polysaccharide vaccine (23vPPV) [1]. Introduction of the vaccine led to decreased rates of hospitalization and death for the Australian population [7]. It has also decreased rates of IPD by up to 93 % in vaccinated children <5 years old, but protection from OM remains poor in both adults and children and causes significant morbidity and mortality [8–11].

## Case presentation

A 53-year-old, Caucasian female, with no significant medical history and normal body mass index (19.6 kg m^–2^), presented to the Emergency Department in a regional Australian hospital with 2 days of sudden-onset left shoulder pain and exacerbation of long-standing lower back pain. This was accompanied by generalized mild myalgias and subjective fevers. The patient had an X-ray of the shoulder showing no fractures and was discharged with a diagnosis of musculoskeletal-related pain with secondary respiratory viral infection, for follow-up with her general practitioner.

Five days later, the patient was brought in by ambulance, with 2 days of confusion and sudden-onset hallucinations. Her shoulder had significant swelling with decreased range of movement and her right hand was swollen and painful. The patient had increased back pain and substantial paraspinal tenderness and increased urinary frequency. The patient also complained of reduced left-sided hearing. Examination with an otoscope revealed a thickened tympanic membrane, mild erythema of the canal and mastoid tenderness. Initial investigations demonstrated an elevated white cell count (WCC) of 29.3×10^9^ l^−1^ (reference range 4.5–11.0×10^9^ l^−1^), of which 26.6×10^9^ l^−1^ were neutrophils, and high inflammatory markers with a C-reactive protein (CRP) of 254 mg l^−1^ (reference range <5 mg l^−1^) [12, 13]. Blood and urine cultures were taken. Her initial imaging, a computed tomography (CT) of the brain and a chest X-ray, were unremarkable. The working diagnosis was septic arthritis or pyelonephritis.

On day 1 of her admission, her blood cultures were flagged as positive for presumptive pneumococcus. In the absence of a validated pneumococcal PCR within a reasonable turnaround time, a small volume of blood culture broth was tested using a BinaxNOW *Step. pneumoniae* antigen card, which yielded a positive result. Empirical intravenous (IV) antibiotic therapy was started with high-dose benzylpenicillin (2.4 g 4-hourly) and ceftriaxone 2 g twice daily. Urinary antigen and multiple peripheral blood cultures were confirmed to be positive for *S. pneumoniae* within the next 24 h. *S. pneumoniae* was identified by typical colony morphology, Gram stain and optochin susceptibility as shown in Fig. 1. Antibiotic susceptibility testing was performed using the Vitek-2, P-576 panel. Penicillin and ceftriaxone MICs were <0.25 µg ml^−1^ [14]. These results were confirmed by the E strip (Epsilometer) test method. The bacterium also tested as susceptible to moxifloxacin (MIC <0.25 µg ml^−1^), rifampicin (MIC <0.25 µg ml^−1^) and vancomycin (MIC <1 µg ml^−1^) with only intermediate resistance to cotrimoxazole (MIC <20 µg ml^−1^) by the Vitek-2 panel. Subsequently, this was identified to be *S. pneumoniae* serotype 15B.

**Fig. 2.**
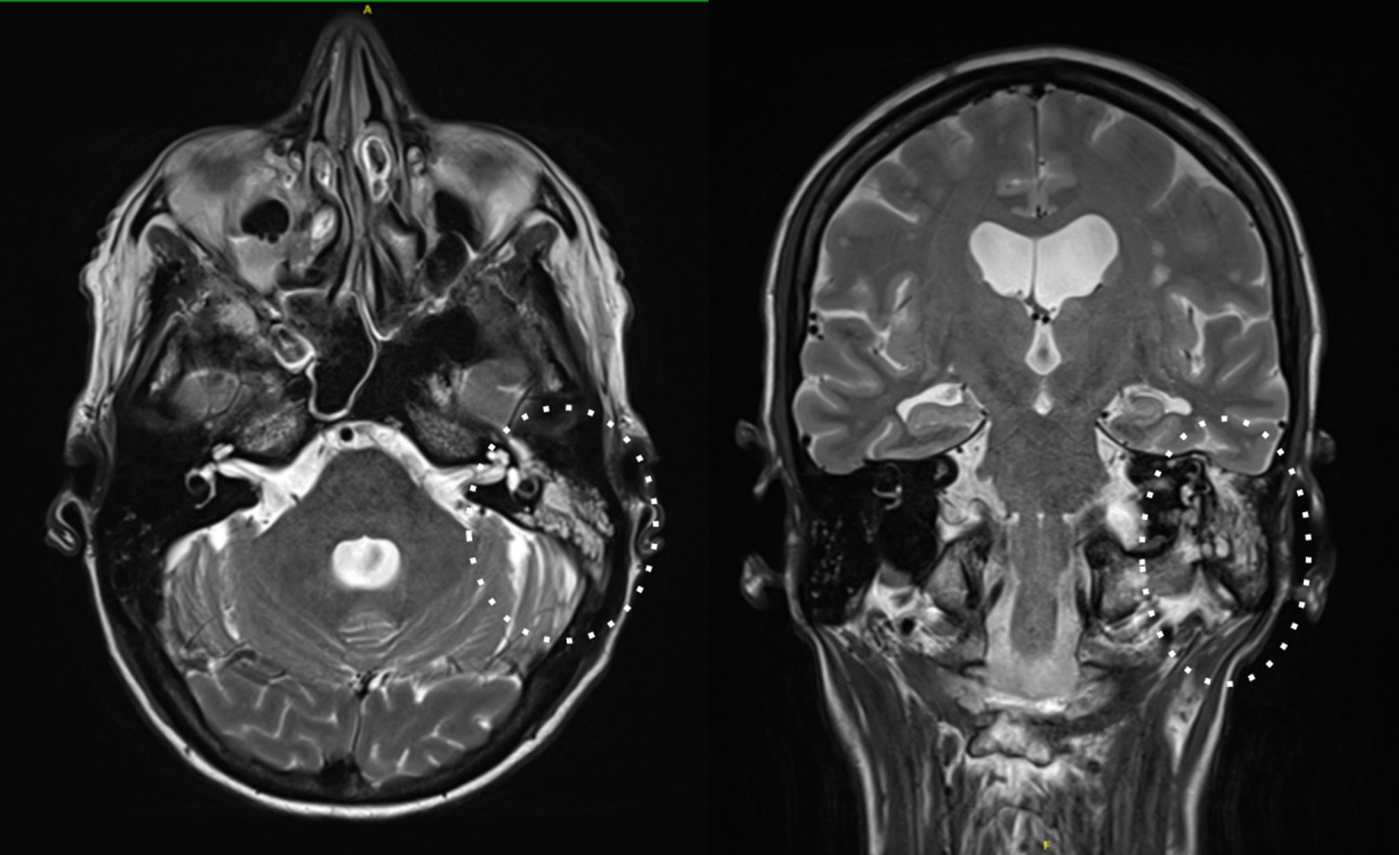
Left otomastoiditis on the patient’s MRI brain scan.

**Fig. 3.**
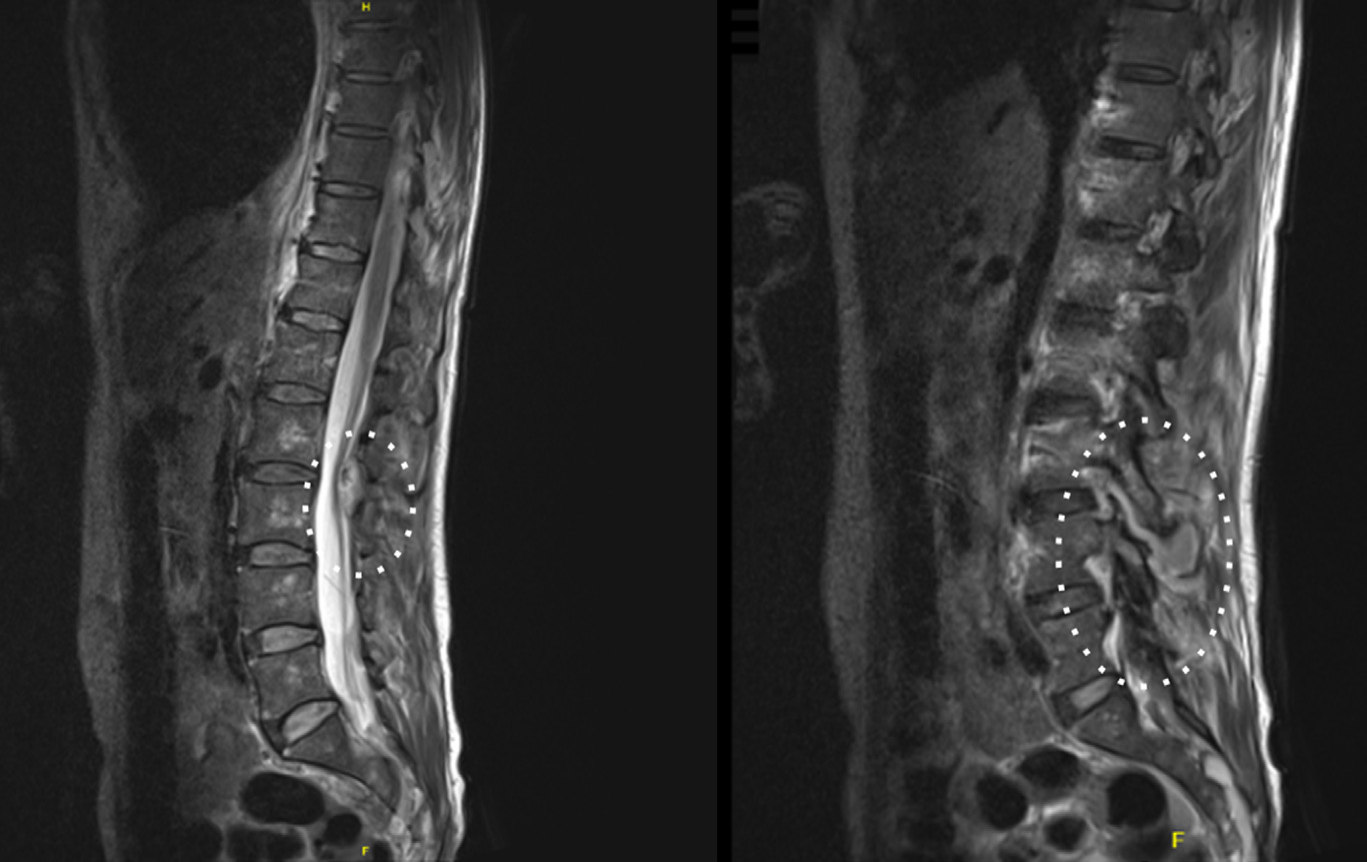
Facet joint septic arthritis and radiological spinal cord compression shown on the patient’s MRI spine scan taken in the acute phase of the disease.

Magnetic resonance imaging (MRI) of the brain and spine demonstrated left mastoiditis and bilateral maxillary sinusitis but showed no evidence of an intracranial abscess or meningeal enhancement (Fig. 2). There was right L2–4 and left L3–5 facet joint septic arthritis with bilateral posterior paraspinal intramuscular abscesses and posterior epidural abscesses from L2–S1 level and into the left L5/S1 foramen. T11/12 discitis was evident with radiological evidence of spinal cord compression (Fig. 3). The radiological abnormalities were discussed with the neurosurgical team at the consulting metropolitan hospital who recommended conservative management under the guidance of an infectious diseases team.

Within 48 h of representation, the patient underwent drainage of the identified right hand and left shoulder effusions, initially under interventional radiology for diagnostic purposes and then as a wash-out in operating theatres for source control. There was evidence of pus within the joints which also grew *S. pneumoniae *. The patient had a tympanostomy tube inserted to allow drainage of pus from the middle ear canal and was started on 10 days of topical ciprofloxacin, in addition to her IV antibiotics. The patient was admitted post-operatively to the intensive care unit (ICU) for management.

On further examination, a systolic murmur of grade 2/6 was identified at the apex of her heart. A cardiac transthoracic echocardiogram (TTE) demonstrated a probable native mitral valve vegetation, and endocarditis was later confirmed on day 2 of her admission, with a transoesophageal echocardiogram (TOE) demonstrating a small vegetation on the anterior leaflet of the mitral valve with mild–moderate incompetence.

Optimum antibiotic treatment for the disseminated penicillin-susceptible pneumococcal infection with neurological involvement was determined to be benzylpenicillin IV 1.8 g 4-hourly accompanied by ceftriaxone IV 2 g, twice daily for the first 48 h. On this regimen, the patient improved in her cognition (attention and orientation), and inflammatory markers were trending downward; by day 3 her CRP was 93 mg l^−1^ and WCC was 22.1×10^9^ l^−1^.

The patient developed abscesses within her left wrist and foot, requiring incision and drainage on day 3. Although the patient was discharged on day 11, she required readmission less than 24 h later due to fevers, increasing back pain and inflammatory markers rising from CRP 121 to 168 mg l^−1^ and WCC from 9.7 to 15.3×10^9^ l^−1^. Repeat imaging showed unchanged disease in the spine except for significant reduction in the size of the epidural collections. No other collections were identified, and urine and blood cultures remained negative. The patient continued the same regimen of antibiotics and the symptoms subsided; no exact cause was identified. The patient was cleared for discharge 3 days later, on day 14 of her original admission.

The patient was discharged with the following plan: (1) benzylpenicillin IV 10.8 g 24-hourly via a peripherally inserted central catheter for 6 weeks from first negative blood culture; (2) monitoring with a weekly full blood count and CRP; (3) TTE 4 weeks after her initial presentation to assess the size of the vegetation; (4) MRI 4 weeks after initial imaging for progress; (5) daily neurological examination under the Hospital in the Home Team (HITH) with any abnormalities escalated to the neurosurgical team; and (6) infectious diseases and cardiology follow-up. Upon consultation with the public health unit, the patient was advised the following immunization schedule of the pneumococcus vaccine: a dose of 13vPCV at diagnosis, dose of 23vPPV 12 months thereafter and second dose of 23vPPV in 5 years.

At 3 months the patient was reviewed, and it was determined that she had no ongoing functional limitations or medical complications. Her follow-up TOE was normal, and the patient was determined to have no ongoing cardiac concerns. The patient completed 6 weeks of antibiotic therapy and had no further fevers, weight loss, pain or abnormal symptoms on cessation. Her inflammatory markers remained low (WCC 6.6×10^9^ l^−1^ and CRP <3 mg l^−1^) on serial testing over 2 months. Multiple investigations were completed, including human immunodeficiency virus (HIV), hepatitis B and C screening, T and B subsets, immunoglobulin levels and subclasses, C3 and C4 levels, serum protein electrophoresis, cryoglobulin detection, and antibody screening (antinuclear, extractable nuclear, dsDNA and antineutrophil cytoplasmic antibodies), all of which were unremarkable. The patient had a body mass index within normal limits, with no nutritional deficiencies able to be detected. Her spleen was *in situ*, confirmed on her abdominal CT. However, functional asplenia cannot be excluded as very few Howell–Jolly bodies were present. Finally, the patient had no family history of immunological deficiencies. Given there was no identifiable cause for this patient’s severe illness, she has been referred for specialist genetic and rheumatology follow-up. The patient in this case study gave explicit written consent.

## Discussion

We describe a case of severe disseminated pneumococcal disease in an immunocompetent woman. Moreover, her clinical picture was complicated by uncommon sites of infection, including endocarditis and septic arthritis. Fortunately, the patient made a full recovery; this case highlights that IPD remains a severe disease and should be considered in an individual with sepsis. Moreover, in the right clinical context and in the presence of multiple positive microbiology samples, point of care diagnostic tools available in this regional laboratory allowed for prompt diagnosis and early directed therapy.

IPD is a notifiable disease in Australia [15]. In August 2022, NSW health reported 232 cases of IPD thus far in 2022, which was higher than the totals for the previous two years (251 in 2021 and 170 in 2020) [16]. Most cases occur in extremes of ages (<2 years old or >85 years old) and in Aboriginal and Torres Strait Islanders [1]. This was an unexpected presentation, as our patient was a middle-aged, Caucasian female with no underlying factors to predispose her to severe disease [17].

### Vaccination to prevent IPD

The pneumococcal conjugate vaccine was introduced in Australia in 2005 (Prevenar 7) as part of the infant schedule, with no catch up offered to people over 2 years of age without underlying risk factors for severe disease, and hence the patient did not qualify for a National Immunization Programme-funded vaccine. The Australian Immunization Handbook outlines the goal of herd immunity via the targeted vaccination strategy – reduced carriage of *S. pneumoniae* as a microbial flora in the general population due to vaccination from a young age [1]. There is good uptake of the vaccine in Australian infants (up to 94 %), but uptake of the adult schedule continues to remain poor in the elderly (as low as 36 % in eligible adults) [7, 18]. However, with a history of IPD, this patient now qualifies for a funded vaccine schedule to protect against other pneumococcal serotypes.

In this case, IPD developed from the pneumococcal serotype 15B. This is significant as serotypes 15A, 15B and 15C are the serotypes most associated with IPD since the introduction of the 13vPCV. Serotype 15B also contributes significantly to the rates of OM, second only to serotype 6 as the identified causative serotype, as was the initial source of infection in the patient studied [19, 20]. Notably, serotype 15B is associated with a high fatality rate (within the top five serotypes for increased mortality), meningitis (within the top 10 most common serotypes) and antibiotic resistance compared to other pneumococcus serotypes, and is not covered by the recently Australian registered 15-valent conjugate pneumococcal vaccine (Vaxneuvance) but is present in the 20-valent conjugate pneumococcal vaccine (Prevnar20), not currently registered in Australia [21–24]. The 23vPCV covers for serotype 15B, which early research suggests also provides some limited protection from serotype 15C due to immune cross-protection [19, 25].

### Diagnosis

Routine laboratory diagnosis of pneumococcal sepsis includes aerobic and anaerobic blood cultures, Gram stain and microscopy, and *S. pneumoniae* nucleic acid detection [targeting Lyt A and the pneumolysin (*ply*) gene] [26]. Nucleic acid detection can be done either on EDTA blood or a positive blood culture broth, via a pneumococcal monoplex PCR as a part of multiplex PCR [3, 4, 6, 27]. Pneumococcal antigen has been useful in the diagnosis of meningitis, sepsis and pneumonia. Direct matrix associated laser deionization time of flight (MALDTOF) can be useful to identify pneumococci directly from positive blood culture broth. Additionally, there are several other technologies available such as PCR/electrospray ionization mass spectrometry (ESI-MS), peptide nucleic acid fluorescent in situ hybridization (PNA-FISH), 16S RNA gene sequencing and magnetic resonance-based detection [28–34]. Pneumococcal antigen testing of urine has a low specificity, especially in children as it may represent pneumococcal carriage, but allows for rapid diagnosis and is validated in patient care [30]. We used the antigen test, which is not validated for blood cultures, but it provided us with an early clue as to the cause of sepsis in this individual and allowed us to optimize her management [35].

### Infection

Invasive spread of *S. pneumoniae* most commonly includes the blood, lungs and meninges. Rarer sites of IPD relate to septic arthritis, osteomyelitis and endocarditis [36]. It can also cause disease in obscure sites including peritonitis, ileitis, appendicitis, solid organ abscesses and pericarditis [2].

Septic arthritis affects 1.6 % of patients with IPD. The key risk factors for developing septic arthritis are male sex, inability to walk independently prior to infection and underlying joint disease, none of which were relevant to the patient of this case study [37].

Our patient had endocarditis, and *S. pneumoniae* accounts for less than 1 % of all native valve endocarditis cases and in less than 0.3 % of IPD cases since the advent of penicillin. Pneumococcal endocarditis carries a poor prognosis, with fatality rates of 17–39 % and higher rates of heart failure and shock than non-pneumococcal endocarditis [38–41]. Marrie *et al.* [37] attempted to identify risk factors for the development of endocarditis but failed to find anything statistically significant beyond the current known risk factors for IPD – none of which this patient had [39].

### Immune deficiency

In cases such as the one described above in which an invasive encapsulated bacterial infection has been detected in sterile sites (blood, CSF, joint or pleural fluid), clinicians are advised to investigate for an underlying pre-disposing condition [42–44]. Initially one must obtain information on vaccination status, implanted materials (such as a cochlear implant), IV illicit drug use or pre-existing conditions, such as CSF leak, Down syndrome, congenital heart disease or renal failure [39, 43].

Immunodeficiency must be considered, both primary and secondary. Secondary immunodeficiencies include immunosuppressing medications, human immunodeficiency virus (HIV) infection and autoimmune diseases, such as type 1 diabetes. A family history of immunodeficiencies should also be screened when the history is taken [43, 45].

If all the above risk factors are negative, then further investigations must be completed. Initially blood pathology should be completed, including full blood count (FBC) and manual blood film examination for Howell–Jolly bodies (>1 % pathognomonic for splenic dysfunction), IgG, IgM and IgA levels, HIV serology, complement function testing (including absent complement alternative pathway (AH50) and classical complement pathway (CH50)) and mannose binding lectin [42, 43, 46]. Imaging, specifically splenic ultrasound, should be completed to assess spleen size and abnormalities [43, 47].

### Treatment considerations

In IPD, effective, targeted treatment must be started quickly for optimum patient outcomes [48, 49]. In IPD cases without associated meningitis, IV benzylpenicillin is the first-line therapy, but once the pneumococcus has spread to the central nervous system antibiotic selection is more complicated [48, 50]. Rates of antimicrobial resistance (AMR), especially to penicillin, continue to be a concern [51]. Therefore, penicillin MICs need to be determined accurately. Three key known penicillin-binding proteins as well as many unknown mechanisms determine the exact MIC of the isolate of the patient [52]. The Australian Therapeutic Guidelines (2022) recommend adjusting the antibiotic regimen depending on the level of MIC identified. For pneumococcal isolates with a penicillin MIC <0.125 µg l^−1^, benzylpenicillin remains first line, but for a penicillin MIC ≥0.125 µg l^−1^, that is strains resistant to penicillin, ceftriaxone or cefotaxime are recommended. For strains which are penicillin-resistant (MIC >0.125 µg l^−1^) and cephalosporin-resistant (MIC 1.0–2.0 µg l^−1^) then vancomycin or moxifloxacin are added to the regimen [48, 49]. Hence, careful determination of accurate MICs is key in achieving targeted treatment [49].

## Conclusion

This was an interesting case in which a common, preventable pathogen led to a life-threatening disease. Our patient had no risk factors for developing IPD, but consideration of this disease leading to fast diagnosis and treatment was key in achieving good outcomes for the patient. While vaccination for *S. pneumoniae* is improving immunity, many populations remain unvaccinated, and AMR continues to develop, which is creating challenges for fast and effective treatment. Hence, IPD must continue to be part of our differential diagnosis in patients from any population.
